# Efficacy of Biorational Products for Managing Diseases of Tomato in Greenhouse Production

**DOI:** 10.3390/plants11131638

**Published:** 2022-06-21

**Authors:** Luis Fernando Esquivel-Cervantes, Bertha Tlapal-Bolaños, Juan Manuel Tovar-Pedraza, Oscar Pérez-Hernández, Santos Gerardo Leyva-Mir, Moisés Camacho-Tapia

**Affiliations:** 1 Departamento de Parasitología Agrícola, Universidad Autónoma Chapingo, Texcoco 56230, Mexico; luis-esqcer1@hotmail.com (L.F.E.-C.); lsantos@correo.chapingo.mx (S.G.L.-M.); 2 Laboratorio de Fitopatología, Coordinación Regional Culiacán, Centro de Investigación en Alimentación y Desarrollo, Culiacán 80110, Mexico; 3 Department of Plant Sciences and Plant Pathology, Montana State University, Bozeman, MT 59717, USA; oscar.perezhernandez@montana.edu; 4 Laboratorio Nacional de Investigación y Servicio Agroalimentario y Forestal, Universidad Autónoma Chapingo, Texcoco 56230, Mexico; moises.camachotapia@gmail.com

**Keywords:** *Solanum lycopersicum*, integrated disease management, plant extracts, defense inducers, incidence, AUDPC

## Abstract

Gray mold (*Botrytis cinerea*), late blight (*Phytophthora infestans*), powdery mildew (*Leveillula taurica*), pith necrosis (*Pseudomonas corrugata*), and bacterial canker (*Clavibacter michiganensis*) are major diseases that affect tomato (*Solanum lycopersicum* L.) in greenhouse production in Mexico. Management of these diseases depends heavily on chemical control, with up to 24 fungicide applications required in a single season to control fungal diseases, thus ensuring a harvestable crop. While disease chemical control is a mainstay practice in the region, its frequent use increases the production costs, likelihood of pathogen-resistance development, and negative environmental impact. Due to this, there is a need for alternative practices that minimize such effects and increase profits for tomato growers. The aim of this study is to evaluate the effect of biorational products in the control of these diseases in greenhouse production. Four different treatments, including soil application of *Bacillus* spp. or *B. subtilis* and foliar application of *Reynoutria sachalinensis*, *Melaleuca alternifolia*, harpin αβ proteins, or bee honey were evaluated and compared to a conventional foliar management program (control) in a commercial production greenhouse in Central Mexico in 2016 and 2017. Disease incidence was measured at periodic intervals for six months and used to calculate the area under the disease progress curve (AUDPC). Overall, the analysis of the AUDPC showed that all treatments were more effective than the conventional program in controlling most of the examined diseases. The tested products were effective in reducing the intensity of powdery mildew and gray mold, but not that of bacterial canker, late blight, and pith necrosis. Application of these products constitutes a disease management alternative that represents cost-saving to tomato growers of about 2500 U.S. dollars per production cycle ha^−1^, in addition to having less negative impact on the environment. The products tested in this study have the potential to be incorporated in an integrated program for management of the examined diseases in tomato in this region.

## 1. Introduction

Tomato (*Solanum lycopersicum* L.) is one of the most widely cultivated plants in the world and is appreciated for its high nutritional value [1]. Mexico is the ninth largest producer, with 3.4 million tons annually, and the largest exporter of tomato worldwide [2]. Out of the total production of tomato in Mexico, 40% is conducted in greenhouses. A major constraint in greenhouse tomato production is diseases, several of which occur each season. Among the most frequent diseases are those caused by fungi, oomycetes, and bacteria; damage is reported in pre- and postemergence, transplanting, and crop development [3,4]. The most concerning diseases in this region are caused by oomycetes such as *Pythium aphanidermatum* and *Phytophthora capsici*, and by fungi such as *Rhizoctonia solani*, *Fusarium oxysporum, F. solani*, *Verticillium dahliae*, *Macrophomina phaseolina*, *Botrytis cinerea*, *Sclerotinia sclerotiorum,* and *Sclerotium rolfsii*. During crop development, it is common to find *Phytophthora infestans*, *Botrytis cinerea*, *Alternaria solani*, *Sclerotinia sclerotiorum*, *Phoma lycopersici*, *Septoria lycopersici,* and *Leveillula taurica.* Bacterial diseases caused by the species *Clavibacter michiganensis*, *Pseudomonas corrugata*, *Pseudomonas syringae* pv. *tomato*, *Xanthomonas vesicatoria,* and *Pectobacterium carotovorum* [5] also affect the crop in the growing season. All of these diseases cause damage to roots and above-ground parts, including fruit, and inflict high economic losses [5], mainly due to their cost of control. Virtually, the only way to control these diseases is with the use of chemical products. Many growers in this region carry out up to 24 applications in a growing season to ensure a harvestable crop. The repeated fungicide applications elevate production costs, leaving growers with small margins that render the operations unsustainable. In addition, the numerous applications have a negative impact on the environment. This latter aspect has become more concerning due to the public pressure and demand for healthy tomato fruit. In view of that, there is an urgent need for alternative sustainable strategies to manage these diseases. A relatively new approach to disease control in the region is the use of biorational products. Biorational products are low-environmental-impact products for use in agriculture [6,7,8]. They include biopesticides (fungi or bacteria), botanicals (plant extracts, oils), minerals, as well as products for crop stress management [6,7]. Several products are of commercial use at present, for example, a large number of bacterial strains have been isolated and identified as biocontrol agents against tomato diseases [8]. Among them, the genus *Bacillus* is widely distributed and recognized as an effective biocontrol agent of crop diseases [9]. *Bacillus*-based biological control agents (BCAs) have great potential in integrated pest management (IPM) systems; however, most research has focused on BCAs as alternatives to synthetic chemical fungicides or bactericides and not as part of an integrated disease management system [10]. The main mechanisms of action of *Bacillus* spp. are the excretion of antibiotics, toxins, siderophores, and lytic enzymes as well as the induction of systemic resistance [9]. Currently, various commercial formulations have strains of the genus *Bacillus* as an active ingredient, due to their colonization capacity, easy reproduction, and high persistence associated with the formation of endospores, the last being a characteristic of special interest as it allows them to survive under abiotic stress conditions, facilitating their production and storage for long periods of time [11]. The efficacy and performance of these products in commercial tomato production are unknown.

The aim of this study is to evaluate the efficacy of biorational products in controlling diseases in greenhouse tomato production. The ultimate goal is to be able to incorporate these tactics into an integrated disease management system in commercial tomato production.

## 2. Results

**Figure 1:**
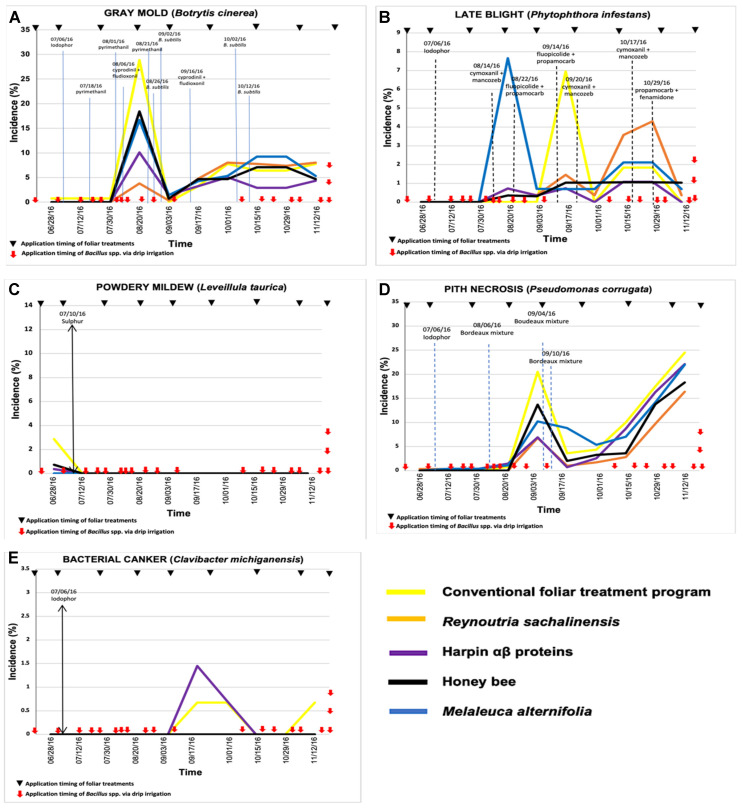
Temporal progress of five major tomato diseases—(**A**) gray mold (*Botrytis cinerea*), (**B**) late blight (*Phytophthora infestans*), (**C**) powdery mildew (*Leveillula taurica*), (**D**) pith necrosis (*Pseudomonas corrugata*), and (**E**) bacterial canker (*Clavibacter michiganensis*)—under the effect of different treatments consisting of soil application of *Bacillus* spp. via drip irrigation and foliar application of biorational products via spraying in greenhouse tomato production in Mexico in 2016. Dotted vertical lines in graphs indicate the approximate timing of application of fungicides in the conventional management program or in any other treatment due to low intensity tolerance to disease. Incidence values are the average of three replications.

**Figure 2:**
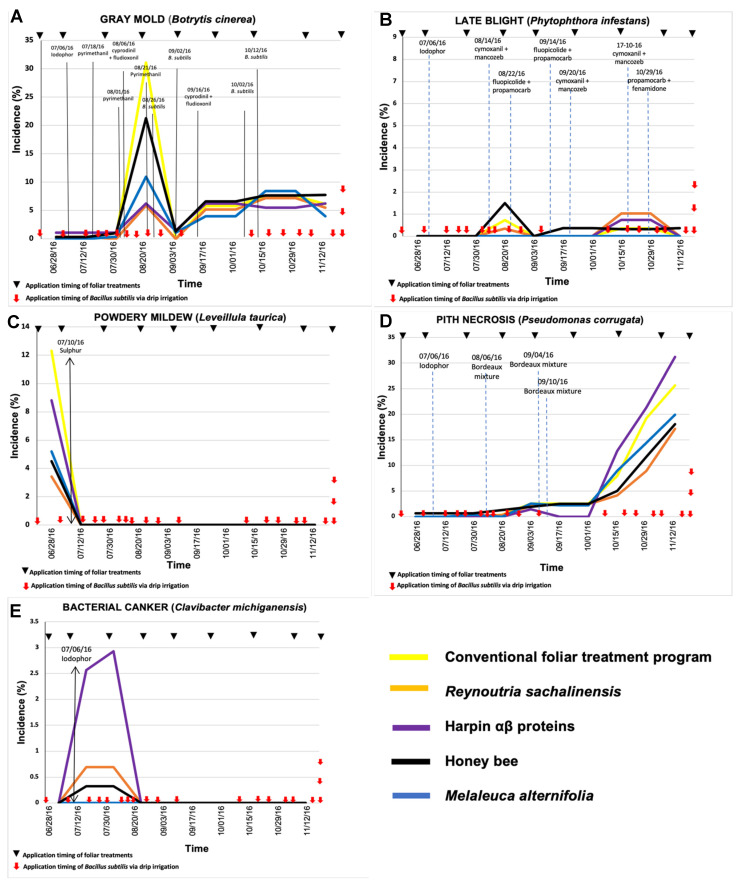
Temporal progress of major tomato diseases—(**A**) gray mold (*Botrytis cinerea*), (**B**) late blight (*Phytophthora infestans*), (**C**) powdery mildew (*Leveillula taurica*), (**D**) pith necrosis (*Pseudomonas corrugata*), and (**E**) bacterial canker (*Clavibacter michiganensis*)—in five different treatment combinations consisting of soil application of *Bacillus subtilis* via drip irrigation and foliar application of biorational products in tomato greenhouse production in Experiment 2 in 2016. Dotted vertical lines in graphs indicate the approximate timing of application of fungicides in the conventional management program or in any other treatment due to low-intensity tolerance to disease. Incidence values are the average of three replications.

**Figure 3:**
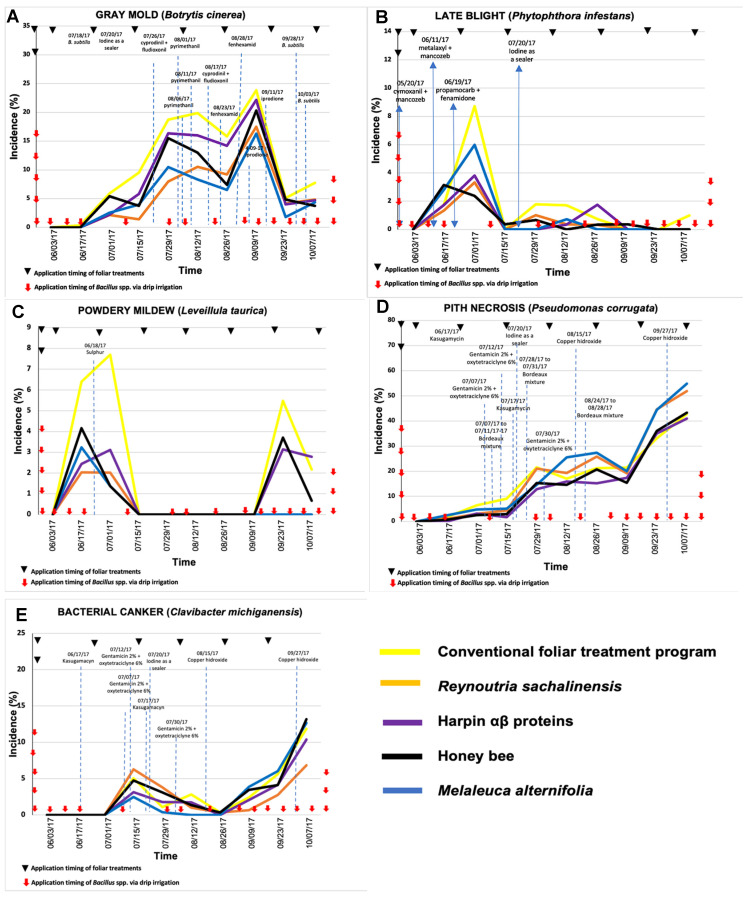
Temporal progress of major tomato diseases—(**A**) gray mold (*Botrytis cinerea*), (**B**) late blight (*Phytophthora infestans*), (**C**) powdery mildew (*Leveillula taurica*), (**D**) pith necrosis (*Pseudomonas corrugata*), and (**E**) bacterial canker (*Clavibacter michiganensis*)—in five different treatment combinations consisting of soil application of *Bacillus* spp. Via drip irrigation and foliar application of biorational products in tomato greenhouse production in Experiment 1 in 2017. Dotted vertical lines in graphs indicate the approximate timing of application of fungicides in the conventional management program or in any other treatment due to low-intensity tolerance to disease. Incidence values are the average of three replications.

**Figure 4:**
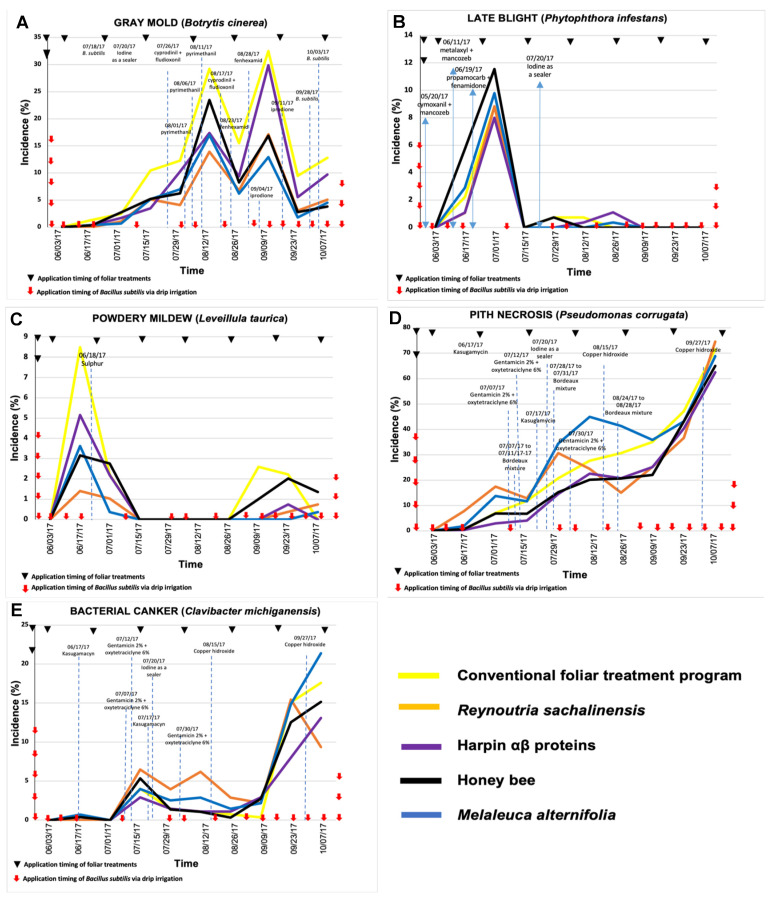
Temporal progress of major tomato diseases—(**A**) gray mold (*Botrytis cinerea*), (**B**) late blight (*Phytophthora infestans*), (**C**) powdery mildew (*Leveillula taurica*), (**D**) pith necrosis (*Pseudomonas corrugata*), and (**E**) bacterial canker (*Clavibacter michiganensis*)—in five different treatment combinations consisting of soil application of *Bacillus subtilis* via drip irrigation and foliar application of biorational products in tomato greenhouse production in Experiment 2 in 2017. Dotted vertical lines in graphs indicate the approximate timing of application of fungicides in the conventional management program or in any other treatment due to low-intensity tolerance to disease. Incidence values are the average of three replications.

In 2016, the lowest incidence values of diseases caused by *B. cinerea* and *L. taurica* were observed with soil-applied *Bacillus* spp. (Figure 1), while the lowest incidence values for late blight and pith necrosis occurred in the treatments with *B. subtilis* (Figure 2). However, no difference was observed in the incidence of bacterial canker between treatments based on *Bacillus* spp. and *B. subtilis*. In 2017, the lowest incidence of the disease caused by *B. cinerea* was observed in tomato plants treated with *B. subtilis*; however, for the other diseases, the lowest incidence values occurred in plants treated with soil-applied *Bacillus* spp. (Figure 3 and Figure 4).

### 2.1. Treatment Effect in 2016: Experiment 1

Gray mold. The tomato plants that received the biorational foliar treatments resulted in numerically low AUDCP compared to the conventional treatment program. Treatment with harpin proteins and *R. sachalinensis* exhibited the highest level of disease control, as indicated by the low AUDPC (Table 1). However, the difference was not significant (Table 1). All treatments indicated a lower incidence of disease than the base-conventional treatment. In terms of incidence, the end-of-season incidence of gray mold showed a significant difference among treatments on August (*P =* 0.0025). The treatments with the lowest incidence of gray mold in this year were those including *R. sachalinensis* and harpin αβ proteins with 3.77 and 10.11%, respectively, while the highest incidence occurred in the nontreated plants with 20.82%. In the rest of the growing season, the difference was not significant (*P* > 0.05) and only a numerical difference was observed, with the best treatment being that of harpin proteins, with incidence below 5% (Figure 1A). Peaks of incidence were more evident through midseason with disease going up and down (Figure 1).

Late blight. Incidence of late blight in the experiment with *Bacillus* spp. (Figure 1B) did not show a significant statistical difference on the dates between treatments, but it did show a numerical difference, where the highest incidence was in August for the treatment with *M. alternifolia*, in September for the control, and from October onwards in the treatment with *R. sachalinensis*, while the lowest incidence during these months occurred in the treatments with harpin αβ proteins and bee honey. The AUDPC values in the experiment with *Bacillus* spp. did not exhibit a statistical difference, but a numerical difference (Table 1), where the lowest was in the treatments with harpin proteins and bee honey with 3.98 and 5.36, respectively. The highest value was not recorded in the nontreated control (10.62), but in the treatment with *M. alternifolia* (14.31).

Powdery mildew. As the season progressed, incidence of powdery mildew in the experiment with *Bacillus* spp. (Figure 1C) showed no statistical difference and only presented incidence at the beginning of the growing season, which was in June in the nontreated control with 2.8% against *M. alternifolia* with 0%. The AUDPC values for powdery mildew (Table 1) showed no significant difference either, where the highest value being 1.43 for the nontreated plants and the lowest (0.0) for the treatment with *M. alternifolia*.

Pith necrosis. Incidence of pith necrosis (Figure 1D) in the experiment with *Bacillus* spp. showed a statistical difference between treatments on 17 September (*P* = 0.013) and 1 October (*P* = 0.034). The treatments with the lowest incidence on 17 September were bee honey (0.72%), harpin proteins (0.99%), *R. sachalinensis* (2.01%), and the nontreated control (3.36%), while the most affected was *M. alternifolia* (8.83%). In October, the best was *R. sachalinensis* with 1.7% and the most damaged was *M. alternifolia*. The AUDPC values for *Bacillus* spp. did not show statistical differences, only numerical (Table 1), where the best treatment was *R. sachalinensis* (30.65) followed by bee honey (45.52).

Bacterial canker. The temporal progress of bacterial canker in the experiment with *Bacillus* spp. (Figure 1E) had an incidence of less than 1.5% in all treatments and there were only outbreaks of this disease in the treatment based on harpin proteins; however, they were so low that they did not represent a statistical difference with the other treatments. The AUDPC values for bacterial canker in the *Bacillus* spp. experiment showed no differences (Table 1).

### 2.2. Treatment Effect in 2016: Experiment 2

The incidence of gray mold in the experiment with *B. subtilis* (Figure 2A) presented a statistical difference and corresponded to the same date (*P* = 0.0020) as in the experiment with *Bacillus* spp., where the highest incidence was reported in the nontreated plants with 31.08% and the lowest in the treatments with *R. sachalinensis* (5.8%) and harpin proteins (6.24%). The most favorable conditions for *B. cinerea* occurred in August in the two trials; so, conventional fungicides were applied preventively and curatively when the incidence exceeded 5%. In the AUDPC values for gray mold, there was no statistical difference, as the treatment with *R. sachalinensis* reached 33.81, which was the lowest value, followed by the treatments with harpin proteins and *M. alternifolia*, with 37.08 and 39.17, respectively (Table 2).

As the season progressed, disease incidence of late blight caused by *P. infestans* in the experiment with *B. subtilis* (Figure 2B) only exhibited a numerical difference, where the highest incidence was in August with bee honey with 1.49% and the best treatments were *M. alternifolia* and harpin proteins with 0%; in October, the highest incidence was observed in the treatment with *R. sachalinensis*, while the lowest incidence in the whole growing season was in *M. alternifolia* with 0%. For the experiment with *B. subtilis*, there was also no difference among treatments with respect to the AUDPC values for late blight since this presented very low levels (0 to 3.5) in all treatments (Table 2).

The incidence of powdery mildew (Figure 2C) showed a difference on June 28 (0.04%), between the control (12.31%), *R. sachalinensis* (3.43%), bee honey (4.51%), and *M. alternifolia* (5.19%). In addition, for the AUDPC data (Table 2), there was a statistical difference (0.046), and the best treatments for powdery mildew were *R. sachalinensis*, bee honey, and *M. alternifolia* with values of 1.71, 2.25, and 2.59, respectively

The incidence of pith necrosis did not differ among the treatments evaluated and the disease remained below 5% (Figure 2D). However, it was in October that the incidence increased, being the lowest in the treatment with *R. sachalinensis* (17.15%), whereas the highest levels were found in the plants treated with harpin proteins (31.21%). The development of the disease caused by *P. corrugata* according to the temporal dynamics was favored from October, where the incidence increased without decreasing again; this damage is related to the age of the plant in the last months of production. In the case of the AUDPC values (Table 2), there was no statistical difference, but there was a numerical difference, and it was observed that all treatments had a similar effect; however, the lowest AUDPC values were observed in the treatments with *R. sachalinensis* (28.92) and bee honey (35.24), while the highest AUDPC value was in the treatment with harpin proteins (51.21).

The bacterial canker in the experiment with *B. subtilis* (Figure 2E) had an incidence of less than 3% in all treatments and there were only outbreaks of this disease in the treatment with harpin proteins; however, they were so low that they did not represent a statistical difference with the other treatments. The AUDPC data for bacterial canker in the *B. subtilis* experiment (Table 2) also reported no differences among treatments.

### 2.3. Treatment Effect in 2017: Experiment 1

Disease incidence of gray mold in the experiment with *Bacillus* spp. (Figure 3A) showed a significant statistical difference on 12 August (0.022), 26 August (0.019), 9 September (0.042), and 23 September (0.009). The treatments with the lowest incidence on August 12 were *R. sachalinensis* (13.90%), *M. alternifolia* (17.02%), and harpin proteins (17.38%). Noteworthily, for the dates of 26 August as well as September 9 and 23, the best treatment was with *M. alternifolia*. On the other hand, the AUDPC values for gray mold indicated a statistical difference (0.0074), where it was observed that the best foliar treatments were the plant extracts based on *M. alternifolia* and *R. sachalinensis* (Table 1).

The incidence of late blight in the experiment with *Bacillus* spp. (Figure 3B) showed no significant statistical difference in dates among treatments but it did show a numerical difference. The highest values were in July in the control with 8.71% and the lowest in bee honey (2.37%), whereas in the rest of the growing season the values were below 2% in all treatments; so, the numerical difference was minimal. The AUDPC data for late blight (Table 1) did not indicate statistical differences and only had numerical differences, where the best treatment was *R. sachalinensis*.

The incidence of powdery mildew in the experiment with *Bacillus* spp. (Figure 3C) showed a statistical difference on 1 July (*P* = 0.0072), among treatments, where the best were *M. alternifolia*, bee honey, *R. sachalinensis,* and harpin αβ proteins. In the rest of 2017, the levels decreased to 0%, and it was in September when an increase was again observed in the control (5.47%) against 0% in the treatment with *R. sachalinensis*. The AUDPC values for powdery mildew reported not statistical but numerical differences (Table 1), where the best treatments were *R. sachalinensis* and *M. alternifolia*.

The disease incidence of pith necrosis in the experiment with *Bacillus* spp. (Figure 3D) showed a statistical difference on 29 July (*P* = 0.0054) and 7 October (*P* = 0.0212). The best treatment for the control of pith necrosis was with the treatment based on harpin proteins, since it recorded the lowest values throughout the growing season. The AUDPC data for pith necrosis did not present statistical differences and all treatments had the same effect (Table 1), but the lowest AUDPC was for the treatment with harpin proteins.

The incidence of bacterial canker (Figure 3E) showed no statistical difference between dates in the treatments, but it did show a numerical difference. Throughout the growing season, there was an increase in mid-July in the treatment with *R. sachalinensis* and the lowest incidence was in the treatment with *M. alternifolia*. The AUDPC data for bacterial canker also showed no differences among treatments (Table 1).

### 2.4. Treatment Effect in 2017: Experiment 2

In 2017, the experiment with *B. subtilis* for gray mold control (Figure 4A) showed a statistical difference on 29 July (*P* = 0.046) and 26 August (*P* = 0.024), among treatments. The treatment with the lowest incidence was that of 29 July with *R. sachalinensis* (7.97%); however, for 26 August, the best was with *M. alternifolia* (6.52%). The most favorable conditions for the disease caused by *B. cinerea* were from July to September according to the temporal dynamics. For the AUDPC values of gray mold (Table 2), statistical differences were also shown (*P* = 0.035) and the best treatments were *M. alternifolia* and *R. sachalinensis.*

In the experiment with *B. subtilis* for late blight control, no statistical difference was found among treatments, only numerical, and the highest incidence occurred in July in the treatment with bee honey (11.53%) and the lowest in the treatment with harpin αβ proteins (8.0%) compared with the untreated control (8.80%). In the remainder of 2017, the values were <1%, so there was practically no presence of this disease. Favorable conditions for the development of *P. infestans* were observed in June and July 2017, similar to the conditions that occurred in 2016 (Figure 4B). For the experiment with *B. subtilis*, there was also no significant statistical difference in the AUDPC data (Table 2), since practically all treatments had very similar levels; even so, among these levels, the least affected corresponded to the treatment with *R. sachalinensis*.

In the experiment with *B. subtilis,* there was no statistical difference in the foliar treatments for powdery mildew management (Figure 4C), only numerical, and it was the treatment with *R. sachalinensis* that presented the lowest incidence of the disease. Favorable conditions for the development of *L. taurica* were observed in June 2017 and at the end of September, where environmental conditions were favorable and marked the moment to make the decision to apply the necessary prevention measures. In the experiment with *B. subtilis,* there was a statistical difference (*P* = 0.048) in the AUDPC values (Table 2) and the best treatments were *R. sachalinensis* and *M. alternifolia.*

The temporal progress of pith necrosis in the experiment with *B. subtilis* presented a statistical difference on 15 July (*P* = 0.0139), where the best treatment was with harpin proteins, while in the rest of the growing season, there was no difference among treatments, but the lowest incidence was observed in plants treated with harpin proteins (Figure 4D). The AUDPC data for pith necrosis (Table 2) did not present statistical differences and all treatments had the same effect, but the lowest AUDPC value was for the treatment with harpin αβ proteins.

In the experiment with *B. subtilis,* there was a statistical difference in the incidence values for bacterial canker on 23 September (*P* = 0.0257). According to the temporal progress, the favorable conditions for the development of *C. michiganensis* were observed from July to September (Figure 4E), while the AUDPC values did not show significant differences among treatments (Table 2).

## 3. Discussion

Tomato diseases with the greatest impact in the 2016 and 2017 growing seasons were gray mold, late blight, powdery mildew, pith necrosis, and bacterial canker. The results from the present study showed that *Bacillus* spp. (PHC Colonize^®^) and *B. subtilis* (Fungifree AB^®^) applied as a soil treatment prevented the development of fungal diseases in the root of tomato plants, which are common in the area where the study was conducted. Other studies have reported that *Bacillus* species are a good alternative for the integrated management of soilborne tomato diseases, such as Rhizoctonia or Pythium damping-off, and Fusarium wilt [12,13].

Our study showed that disease incidence caused by *B. cinerea* was significantly reduced with the application of foliar treatments based on extracts of *R. sachalinensis* and *M. alternifolia* in both growing seasons. Previous studies have shown that *M. alternifolia* oil destroys the cell wall and changes the composition of the cell membrane of *B. cinerea* hyphae, increasing its permeability and allowing the release of cellular material [14]; whereas, in the case of *R. sachalinensis,* it has been found that this extract significantly reduced the percentage of *B. cinerea* colonization on strawberry leaves [15]. In addition, the volatile phase of the extracts is known to be more toxic and effective than the contact phase for the control of plant pathogenic fungi [14,16].

According to the incidence of late blight caused by the oomycete *P. infestans*, it was observed that the treatment with harpin αβ proteins presented the lowest values, which has also been reported in previous studies reporting the reduction of the disease caused by *P. infestans* in tomato plants by foliar sprays of harpin proteins [17]. On the other hand, plant extracts based on *R. sachalinensis* and *M. alternifolia* did not satisfactorily reduce the disease caused by *P. infestans*. In this regard, Seidl Johnson et al. [18] found that *R. sachalinensis* extract was ineffective in controlling the disease caused by different isolates of *P. infestans* in tomato. In contrast, Reuveni et al. [19] reported that foliar sprays of *M. alternifolia* at 0.5–1% were effective in prophylactically reducing the disease caused by *P. infestans* under greenhouse conditions.

In relation to the development of powdery mildew caused by *L. taurica*, this disease was favored at the beginning of the growing season; so, preventive fungicide applications should be made prior to an outbreak occurring. In the present study, it was determined that foliar sprays of *M. alternifolia* and *R. sachalinensis* extracts are effective for the management of powdery mildew in tomato plants. Similar results were observed in other studies, where *M. alternifolia* extract was reported to have a good effect in the control of powdery mildew in different crops, including tomato [19] and cucumber [20]. Similarly, the effect of *R. sachalinensis* extract in the control of powdery mildew in tomato has been verified [21,22], as well as in the control of powdery mildew in cucumber [23,24], where foliar sprays significantly reduced conidial germination and disease severity due to the direct effect on the fungus and the induction of plant defense responses through the formation of callose papillae, hydrogen peroxide accumulation, and induction of the salicylic acid-dependent pathway [25].

In the case of pith necrosis, caused by *Pseudomonas corrugata*, the products evaluated in this study did not control the disease. Tomato pith necrosis appears to develop when there are low night temperatures, high nitrogen levels, and high humidity [1]. According to the temporal progress, favorable conditions for the development of the disease were observed from July and continued until October; therefore, prior to this time is when the necessary preventive measures should be taken, such as applications with copper-based products, local treatment with Bordeaux mixture, and bactericide applications. In addition, it has been recommended to avoid high doses of nitrogen fertilization and regularly sanitize tools such as clippers and pruning shears [1].

Regarding *C. michiganensis,* this plant pathogenic bacterium did not cause catastrophic damage and the disease was kept under control throughout the growing season, which could be related to the application of *Bacillus*. In this regard, Abo-Elyousr et al. [26] recorded that *B. subtilis* and *B. amyloliquefaciens* can successfully decrease bacterial canker disease in tomato plants by producing antibiotics, siderophores, and lytic enzymes. In 2017, the development of bacterial canker was significantly higher compared with that in 2016, which is attributed to the fact that the pathogen was possibly in the seed, given that there are reports indicating that the onset of this disease or primary infection results from internally or superficially infested seeds [27], which leads to carrying out preventive measures, such as those used here, to avoid damage from this or any other problem. On the other hand, it was observed that the application of harpin αβ proteins had no control effect on bacterial canker, coinciding with the findings reported by Ustun et al. [28] in Turkey. Similarly, Obradovic et al. [29] reported that harpin proteins did not induce effective defense responses of tomato plants against the bacterium *Xanthomonas vesicatoria.*

It should be noted that although there were no statistical differences in all the combinations of *Bacillus* with the foliar treatments in this study, there was a numerical difference, which showed important results since the number of diseased plants was reduced. In addition, the lowest values were in the interactions where the *Bacillus* spp.-based product was used.

In summary, there is convincing evidence that the soil and foliar application of the six biorational products tested in this study are effective in controlling potato late blight, gray mold, powdery mildew, pith necrosis, and bacterial canker in greenhouse tomato production compared to the conventional treatment used by growers in this region. The findings in this study contribute to the development of integrated disease management strategies, which include cultural practices aimed at reducing inoculum or avoiding conditions that predispose crops to the development of the disease; the application of biological products; and, ultimately, the use of chemical measures. This implementation in the tomato crop allowed combining natural inducers (bee honey and harpin αβ proteins), plant extracts (*R. sachalinensis* and *M. alternifolia*), beneficial microorganisms (*Bacillus* spp.), and cultural and chemical alternatives, with the last element only being applied when necessary. The products tested in this study can be integrated into this management system; in addition to saving on chemical applications and reducing the negative impact on the environment, they allowed opening up a panorama for integrated crop management (ICM) that can be further refined for a more sustainable use of resources.

## 4. Materials and Methods

### 4.1. Description of the Experiments

Two experiments were conducted in 2016 (and repeated in 2017) in a low-technology 1000-m^2^ greenhouse located in the municipality of Coatepec Harinas, State of Mexico, Mexico. The greenhouse space was divided into two sections of ~500 m^2^ to establish the two experiments. In each experiment, six biorational commercial products (Table 3) were tested in a number of treatment combinations (described in Table 4), each including soil application of either of two *Bacillus*-containing commercial products as a base treatment. The six products that were tested in this study were selected because of their reported effect on diseases in other cropping systems and because of their affordability for growers.

The soil in this greenhouse had an initial pH of 4.8, EC of 0.56, and organic matter content of 5.4. The soil is not fumigated and has a history of occurrence of the major tomato diseases. In addition, the area of study is an area where these diseases are prevalent and expected to occur each year. For the case of potato late blight and gray mold, one or two applications of fungicides were necessary to ensure a harvestable crop. Weed control during both growing seasons was carried out manually.

### 4.2. Experiment 1

In this experiment, a *Bacillus*-spp.-based product (PHC Colonize^®^) was applied to the soil in one of the 500 m^2^ greenhouse sections through a drip irrigation system. Eight days later (28 June 2016), four week-old tomato seedlings cv. Aguamiel (Vilmorin^®^, Paris, France) were transplanted to marked rows inside the greenhouse section. Row spacing was 1.20 m and interplant spacing was 25 cm. The treatments were disposed in a randomized complete block design with three replications. The experimental units consisted of two 10 m-long, 1.20 m-wide rows. Thereafter, application of *Bacillus* spp. to the soil started eight days after transplant and continued for the four first months and then were switched to 15-day intervals for the last two months of the study. Foliar applications also started eight days after transplant but continued at 20-day intervals until the end of the study. Applications were carried out with a backpack sprayer when the plants were <60 cm-tall and with a stretcher power sprayer when the plants were >60 cm-tall. The equipment was calibrated at each application and the water pH was adjusted to 5.5 to 6 and amended with an adherent.

### 4.3. Experiment 2

This experiment was carried out in the other 500-m^2^ greenhouse section. The experiment design and size of experimental units remained the same as in experiment 1. However, the soil treatment in the irrigation system was changed to *Bacillus subtilis* instead of *Bacillus* spp. The foliar treatments were the same as in the previous experiment. Both experiments were repeated in 2017.

### 4.4. Statistical Analyses

The incidence of the five major fungi, oomycetes, and bacteria diseases that occurred on the crop during six months of study was evaluated on each experimental unit at 15-day-intervals. Incidence was calculated with the following formula:

$$PI=\frac{n}{N}\;\times \;100$$

where ***PI*** = Percentage of incidence of the disease, *n* = number of seedlings or plants with symptoms of the disease, and *N* = total number of seedlings or plants in the experimental unit.

With the incidence data (temporal incidence percentages), the area under the disease progress curve (AUDPC) was calculated for each disease using the following formula [30]:

$$\mathrm{AUDPC}=\sum_{i=l}^{n-1}\;\left(\frac{y_{i}+y_{i+l}}{2}\right)(t_{i+l\;}-t_{i})$$

where *n* is the number of assessments, *i* is the time point of observation, *y_i_* is the disease incidence, and *t_i_* is the number of days from the start of the experiment to the measurement date.

The value of the AUDPC for each disease was analyzed with a one-way ANOVA using PROC GLM procedure on SAS version 9.4 (SAS Institute, Cary, NC, USA). The assumption of normality and homogeneity of variance was satisfied in the two trials. Differences between treatments within the trials were further compared by a Duncan test at *P* ≤ 0.05 level. The AUDPC data from each experiment were analyzed separately because the species of *Bacillus* used in the conventional treatment (control) in experiment 1 differed from that in experiment 2. Moreover, given that each experiment was repeated once only, it made more sense to keep the year effect as fixed rather than random.

## Figures and Tables

**Table 1:** Effect of soil-application of *Bacillus* spp. and foliar application of different biorational products in the control of major tomato diseases in greenhouse in Mexico in 2016 and 2017.

Treatment	AUDPC ^a^ Gray Mold		AUDPC Late Blight		AUDPC Powdery Mildew		AUDPC Pith Necrosis		AUDPC Bacterial Canker	
	2016	2017	2016	2017	2016	2017	2016	2017	2016	2017
**T1 ^c^**	59.24 ^ab^	119.67 ^a^	10.62 ^a^	15.16 ^a^	1.43 ^a^	20.65 ^a^	68.90 ^a^	153.03 ^a^	3.03 ^ab^	23.26 ^a^
**T2 ^d^**	36.04 ^bc^	53.65 ^c^	10.94 ^a^	6.35 ^b^	0.18 ^a^	4.05 ^b^	30.65 ^b^	164.30 ^a^	0.0 ^b^	18.31 ^a^
**T3 ^e^**	27.80 ^c^	82.93 ^b^	3.98 ^a^	7.64 ^ab^	0.17 ^a^	10.09 ^ab^	48.43 ^ab^	121.60 ^a^	6.52 ^a^	18.07 ^a^
**T4 ^f^**	48.80 ^ab^	53.41 ^c^	14.31 ^a^	9.51 ^ab^	0.0 ^a^	4.60 ^b^	58.39 ^ab^	171.64 ^a^	0.0 ^b^	19.07 ^a^
**T5 ^g^**	45.03 ^abc^	67.8 ^bc^	5.36 ^a^	7.25 ^ab^	0.35 ^a^	9.57 ^ab^	45.52 ^ab^	129.71 ^a^	0.0 ^b^	23.74 ^a^

^a^ AUDPC—Area under the disease progress curve. ^b^ Mean values followed by different letters in the column indicate significant differences among treatments according to a Duncan test (*P* < 0.05). Value in each cell is from three replications. ^c^ T1—Soil application of *Bacillus* spp. + conventional foliar applications. ^d^ T2—Soil application of *Bacillus* spp. and foliar application of extracts of *R. sachalinensis.* ^e^ T3—Soil application of *Bacillus* spp. via drip irrigation + foliar application of αβ harpins. ^f^ T4—Soil application of *Bacillus* spp. via drip irrigation + foliar application of *Melalueca alternifolia.* ^g^ T5—Soil application of *Bacillus* spp. via drip irrigation + foliar application of bee honey.

**Table 2:** Effect of combined use of *Bacillus subtilis* applied to the drip irrigation and foliar sprays of biorational products for controlling tomato diseases under greenhouse conditions in 2016 and 2017.

Treatment	AUDPC ^a^ Gray Mold		AUDPC Late Blight		AUDPC Powdery Mildew		AUDPC Pith Necrosis		AUDPC Bacterial Canker	
	2016	2017	2016	2017	2016	2017	2016	2017	2016	2017
**T1 ^c^**	61.20 ^ab^	103.03 ^a^	1.45 ^a^	12.5 ^a^	6.16 ^a^	15.49 ^a^	48.05 ^a^	215.93 ^a^	0.00 ^a^	31.55 ^a^
**T2 ^d^**	33.81 ^b^	55.34 ^bc^	2.41 ^a^	9.89 ^a^	1.71 ^b^	3.16 ^b^	28.92 ^a^	207.08 ^a^	3.12 ^a^	41.85 ^a^
**T3 ^e^**	37.08 ^ab^	83.11 ^ab^	1.47 ^a^	10.54 ^a^	4.40 ^ab^	8.06 ^ab^	51.21 ^a^	161.81 ^a^	10.6 ^a^	24.39 ^a^
**T4 ^f^**	39.17 ^ab^	52.17 ^c^	0.00 ^a^	13.04 ^a^	2.59 ^b^	4.16 ^b^	40.74 ^a^	261.23 ^a^	0.00 ^a^	39.31 ^a^
**T5 ^g^**	56.53 ^ab^	72.15 ^bc^	3.08 ^a^	18.01 ^a^	2.25 ^b^	9.60 ^ab^	35.24 ^a^	167.89 ^a^	2.75 ^a^	31.38 ^a^

^a^ AUDPC—Area under the disease progress curve. ^b^ Mean values followed by different letters in the column indicate significant differences among treatments according to Duncan test (*P* < 0.05). ^c^ T1—Soil application of *Bacillus subtilis* + conventional foliar applications. ^d^ T2—Soil application of *Bacillus subtilis* and foliar application of extracts of *R. sachalinensis.* ^e^ T3—Soil application of *Bacillus subtilis* via drip irrigation + foliar application of αβ harpins. ^f^ T4—Soil application of *Bacillus subtilis* via drip irrigation + foliar application of *Melalueca alternifolia.* ^g^ T5—Soil application of *Bacillus subtilis* via drip irrigation + foliar application of bee honey.

**Table 3:** Biorational products tested for control of tomato diseases in the greenhouse trials.

Product; Manufacturer	Active Ingredient	Rate
PHC Colonize^®^, PHC	*Bacillus* spp.	2.0 kg/ha
Fungifree AB^®^, FMC	*Bacillus subtilis*	2.0 kg/ha
Regalia Maxx^®^, FMC	Extract of *Reynoutria sachalinensis*	1.25 L/ha
Messenger Gold^®^, PHC	Harpin αβ proteins	150 g/ha
Timorex Gold^®^, Syngenta	Extract of *Melaleuca alternifolia*	5 mL/L
Bee honey	Bee honey	1 mL/L

**Table 4:** Description of the treatment combinations tested in this study for control of major tomato diseases in greenhouse production in 2016 and 2017.

Treatment ^a^	Description
1	Soil application of *Bacillus* spp. via drip irrigation + foliar application (conventional) of grower’s treatments (Control)
2	Soil application of *Bacillus* spp. via drip irrigation + foliar application of *Reynoutria sachalinensis* at 20-day intervals
3	Soil application of *Bacillus* spp. via drip irrigation + foliar application of αβ harpins at 20-day intervals
4	Soil application of *Bacillus* spp. via drip irrigation + foliar application of *Melalueca alternifolia* at 20-day intervals
5	Soil application of *Bacillus* spp. via drip irrigation + foliar application of bee honey at 20-day intervals

^a^ Corresponds to Experiment 1. For Experiment 2, the base treatment *Bacillus* spp. was changed to *B. subtilis*, but the foliar treatments remained unchanged.

## Data Availability

Not applicable.
